# First Broad Screening of Allelopathic Potential of Wild and Cultivated Plants in Turkey

**DOI:** 10.3390/plants8120532

**Published:** 2019-11-21

**Authors:** Tugba Gonca Isin Ozkan, Emine Akalin Urusak, Kwame Sarpong Appiah, Yoshiharu Fujii

**Affiliations:** 1 Department of Biological Production Science, United Graduate School of Agriculture, Tokyo University of Agriculture and Technology, Fuchu, Tokyo 183-8509, Japan; tugbagonca_ozkan@yahoo.com (T.G.I.O.); ksappiah90@gmail.com (K.S.A.); 2 Faculty of Pharmacy, Department of Pharmaceutical Botany, Istanbul University, Fatih, 34116 Istanbul, Turkey; akaline@istanbul.edu.tr

**Keywords:** allelopathy, plants, growth inhibitory activity, sandwich method, Turkey

## Abstract

Turkey has one of the richest plant diversities in the Mediterranean region. In the current literature, no broad screening has been conducted on the potential allelopathy of plants from Turkey. This study aimed to evaluate the allelopathic activity of a large number of plants from Turkey for the first time and to determine the species with significant plant growth inhibitory potentials by bioassay. Dried samples of different plant parts were collected from local herbalists. The sandwich method was used to evaluate the potential allelopathy of 126 medicinal plants belonging to 55 families. The results of lettuce radicle and hypocotyl growth for 10 and 50 mg sample treatment conformed to normal distribution. Significant inhibition on lettuce radicle elongation with 10 mg sample was observed in 40 species, out of which 27 species showed over 50% inhibitory activity. The results suggested that these species could contain potential inhibitory compounds against lettuce radicle or hypocotyl growth. The calyxes of *Hibiscus sabdariffa* (3.2% of control) and the seeds of *Prunus dulcis* (5.7% of control) showed the most potent growth inhibitory activity on lettuce radicle elongation. The potential plant growth inhibitory effects of these plants, together with the fruits of *Rhus coriaria* and seeds of *Prunus mahaleb,* have been reported in this study for the first time. All these plants are medicinal, and the results hereby presented provide essential information about the allelopathic effects of medicinal plants from Turkey.

## 1. Introduction

Weeds pose a significant threat to cultivated plants, thereby making weed control a challenging issue for sustainable agriculture [1]. Allelopathy is a biological phenomenon that can be observed in many plants that release chemicals into the surrounding environment either from their aerial or underground parts in the form of root exudation, leaching by dews and rains, and volatilization or decaying plant tissue. These compounds released into the environment could affect (inhibitory or stimulatory) the growth and development of other organisms such as weeds, other plants, animals and microorganisms [2,3]. In this process, allelochemicals can act as natural weed inhibitors upon their release from various donor plant species. They are present in different plant parts, including the leaves, barks, roots, root exudates, flowers, seeds, pollens, stems and fruits of plants [4,5,6]. The utilization of these allelochemicals and allelopathic plants can be further explored to reduce the heavy dependence on synthetic herbicides and the risk of environmental toxicity [3,7]. Weeds, among other crop pests, can cause the highest potential crop losses in global crop production [8,9]. Consequently, the utilization of the allelopathic effect of plants for weed control has drawn increasing attention of many weed scientists [3].

The screening by bioassays is essential for identifying plants with allelopathic potential because many plants have an allelopathic activity (especially at high concentrations), but only a few have strong allelopathic properties [4,7]. Laboratory bioassays, field testing, and chemical screening are crucial steps in plant allelopathy [7]. Allelopathic potentials of plants in the field may be predicted by the performance of plants in bioassay [10,11]. The screening of a large number of plants by bioassay might be the first significant step in the investigation of related allelochemicals and their application in weed management. The sandwich method was developed to determine the allelopathic activity through plant leachates [2,12] and has been widely used [2,12,13,14]. The method is reliable and less time-consuming under laboratory conditions that could be used in extensive screening in the identification of potential allelopathic species [2,12,15,16,17]. This study focused on medicinal plants from Turkey, which is one of the countries with the richest plant diversity in the Mediterranean region [18,19]. In the existing literature, there is no large-scale screening of the allelopathic potential of medicinal plants and commonly grown crops in Turkey. Our current study aimed to contribute to the existing literature by filling this knowledge gap to some extent. 

In this research, for the first time, a broad range of plants from Turkey was screened for their potential allelopathic activity by using the sandwich method bioassay. Here, 144 samples supplied from herbalists were examined, which are widespread plants in Turkey.

## 2. Results

The results of the sandwich bioassay of 126 species from 55 different plant families are presented in this report. Table 1 shows an overview of the allelopathic potential of the top 30 plant samples with the highest growth inhibitory potential. The total score, which was defined as a total number of criteria indices (criteria at R_10 mg_, criteria H_10 mg_, criteria at R_50 mg,_ and criteria at H_50 mg_) for a sample, was 15 of * for *H. sabdariffa* and *P. dulcis*, 14* for *P. mahaleb* and *P. harmala* and 13* for *R. coriaria*, and *N. sativa*. The families with the highest number of different plant species and samples examined in this study were Lamiaceae (15 species, 15 samples), Asteraceae (14 species, 14 samples), Rosaceae (11 species, 13 samples), Apiaceae (nine species, 11 samples), and Fabaceae (five species, six samples), Brassicaceae and Malvaceae (five species, five samples), as shown in Table 2. The lettuce seedling radicle and hypocotyl growth after 10 and 50 mg sample treatment of all the species are shown in Table S1.

**Figure 1:**
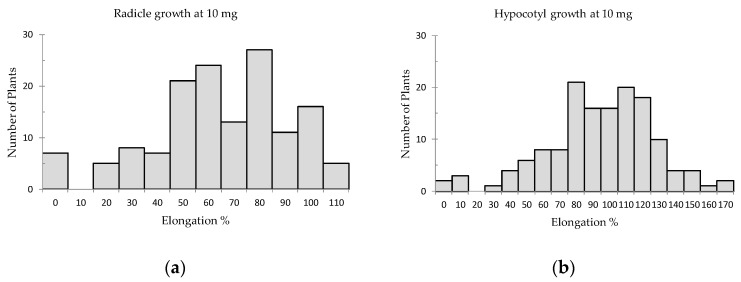
Effect of 10 mg screened samples on lettuce growth with the number of involved species; (**a**) Radicle elongation, (**b**) Hypocotyl elongation.

**Figure 2:**
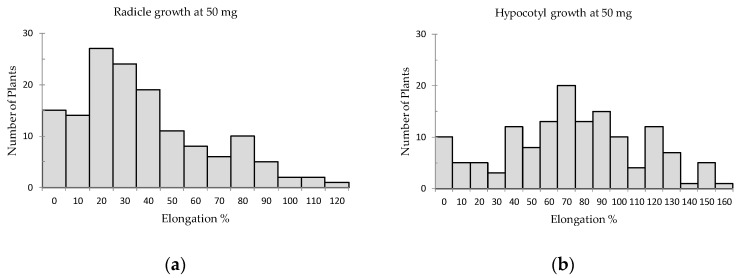
Effect of 50 mg screened samples on lettuce growth with the number of involved species; (**a**) Radicle elongation, (**b**) Hypocotyl elongation.

The results of both lettuce radicle and hypocotyl growth for 10 and 50 mg sample treatment conformed to the normal distribution. The lettuce radicle elongation was affected by potential allelopathic species more than the hypocotyl elongation (Figure 1 and Figure 2). The radicle and hypocotyl elongation percentages of lettuce seedlings were in the range of 3% to 118% and 6.6% to 178%, respectively, for 10 mg sample treatments. The standard deviation (SD) and mean were calculated to assign various criteria for radicle and hypocotyl growths (Criteria at R_10 mg_, Criteria at H_10 mg_, Criteria at R_50 mg_, Criteria at H_50 mg_) to indicate significant inhibition levels among the species as shown in Table S1 and Table 1. Among the 126 plant species, 40 and 39 species respectively had significant inhibition on lettuce radicle and hypocotyl elongation for 10 mg treatments. However, 49 species and 40 species respectively showed significant inhibition on lettuce radicle and hypocotyl when treated with 50 mg.

Among 10 mg treatments of all samples, 27 and 10 of them respectively showed over 50% inhibition on radicle and hypocotyl elongations. *Hibiscus sabdariffa* had less than 4.5% radicle elongation percentage, while six species had radicle elongation between 4.5% and 17.5%, five species between 17.5% and 30.5%, and 28 species between 30.5% and 56.5%. The families with the highest number of species that caused less than 50% radicle elongation for 10 mg treatment were Malvaceae, Asteraceae, Brassicaceae (three species each), Rosaceae (two species), and the rest of the species belonging to 15 other families.

*H. sabdariffa* (Malvaceae) calyxes had the highest inhibitory activity on lettuce seedling elongation. The radicle and hypocotyl elongations were 3.2% and 6.6% of the control respectively, when treated with 10 mg. This was followed by *P. dulcis* (Rosaceae, R_10 mg_% = 5.7%), *T. officinale* (Asteraceae R_10 mg_% = 7.1%), *T. chebula* (Combretaceae, R_10 mg_% = 7.1%), *R. coriaria* (Anacardiaceae, R_10 mg_% = 7.4%), *P. mahaleb* (Rosaceae, R_10 mg_% = 7.8%) and *P. harmala* (Nitrariaceae, R_10 mg_% = 8.6%). 

Five other species (*P. granatum*, *A. rosea*, *L. stoechas*, *S. marianum,* and *N. sativa*) also reduced lettuce seedling radicle elongation in the range of 24.5% to 27.8% of the control. The lettuce radicle elongation in the range of 31.4% to 42.3% of the control was observed for 12 species, while 16 other species reduced seedling elongation in the range of 44.5% to 56.4% of control.

**Figure 3:**
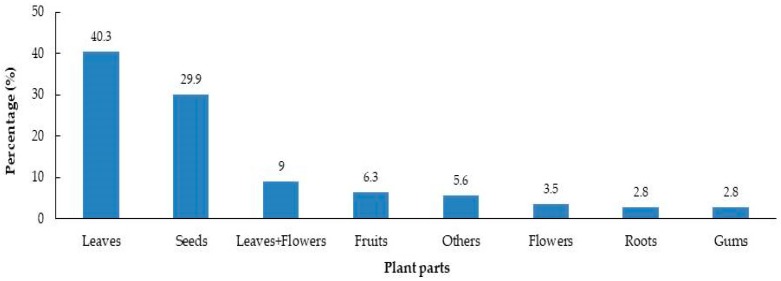
Diversity in plant parts of the evaluated species.

**Figure 4:**
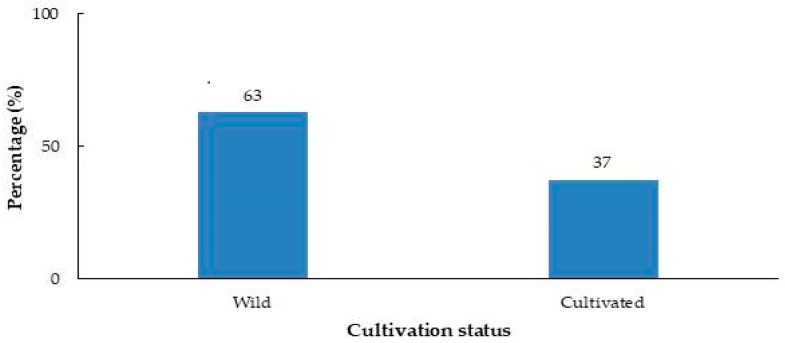
Cultivation status of the medicinal plants evaluated in this study.

Among 50 mg treatments of all samples, 99 and 36 of them respectively showed over 50% inhibition on radicle and hypocotyl elongation. Two species (*P. dulcis* and *L. angustifolia*) had less than 0.6% radicle elongation percentage, while 17 species had radicle elongation between 0.6% and 14.5% and 30 species between 14.5% and 28.4%. The results of this study indicated that increasing dry material weight from 10 to 50 mg enhanced the inhibition of both radicle and hypocotyl growth in lettuce seedlings. The respective mean of radicle elongation percentage for 10 and 50 mg treatment was 69.5% and 42.3%. Leaves (40.3%) were the most used parts of plants in this research, followed by seeds (29.9%), leaves and flowers combined (9%) and fruits (6.3%) in that order (Figure 3). Fifty samples belonged to species broadly cultivated in Turkey, while the rest of the species grow wild in the country (Figure 4). 

## 3. Discussion

In this study, the mean of radicle elongation percentage for 10 mg treatment was 69.5%. The means of radicle elongation percentage for some previous studies were in the range of 60.4% to 71.5% [2,12,13,14]. The mean of radicle elongation percentage for this study was also found in the same range as some other previous studies. Moreover, the standard deviations of radicle elongation percentage for these previous studies were in the range of 20% to 30% for 10 mg treatment. It was seen that the standard deviation (26%) of this current study was also in the same range. The mean of radicle elongation for these previous (805 samples) was calculated as 67.2% for 10 mg treatment (Table 3). Both 10 and 50 mg sample treatment caused stronger inhibition on lettuce radicle elongation than observed in hypocotyl elongation. This observation could be due to the radicle being the first to absorb allelochemicals from the environment. Moreover, root growth has been reported to be more sensitive to phytotoxic compounds than hypocotyl growth [20].

In this research the allelopathic activity of a large number of plants from Turkey was screened and the strong allelopathic species with significant plant growth inhibitory potentials were determined as *H. sabdariffa* (R_10 mg_% = 3.2%, H_10 mg_% = 6.6%), *P. dulcis* (R_10 mg_% = 5.7%, H_10 mg_% = 6.7%), *P. mahaleb* (R_10 mg_% = 7.8% and H_10 mg_% = 11.6%), *P. harmala* (R_10 mg_% = 8.6% and H_10 mg_% = 16.4%) and *R. coriaria* (R_10 mg_% = 7.4%, H_10 mg_% = 33.1%). These plants could potentially contain some chemical compounds, which affected the growth of lettuce seedling upon their release from dry plant samples into the agar medium. In other related studies, the compounds released from the donor plants were responsible for the plant growth inhibitory effect [4,6,11,14,15]. In this section, the chemical information of some previous research and a general introduction of the species with significant plant growth inhibitory potentials will be discussed.

*Hibiscus sabdariffa* L. (Malvaceae), commonly known as Roselle is a widely grown annual plant in tropics and subtropics of both hemispheres and many areas of Central and West Africa, South East Asia, America and elsewhere [21]. *H. sabdariffa* reduced lettuce radicle and hypocotyl elongation to 3.2% and 6.6% of the control, respectively, in this study. The chemical composition of *H. sabdariffa* has been reported to include quercetin, luteolin, chlorogenic acid, protocatechuic acid, pelargonic acid, beta-sitosterol and ergosterol, hydroxy citric acid, delphinidin-3-sambubioside and cyaniding-3-sambubioside in the aqueous extracts [21,22,23,24,25]. Hydroxy citric acid is the principal acid component of the *H. sabdariffa* and was determined to be enriched in the calyxes of *H. sabdariffa* [24]. The red calyx of the plant is used in numerous products, including herbal teas, herbal medicines, syrups and food colouring [26,27,28]. Although the whole plant (leaves, stem and roots) and isolated chemicals from the whole plant, i.e. trimethyl allo-hydroxycitrate and β-sitosterol, showed strong inhibitory activity on the growth of test plant species [20,29,30,31], the allelopathy of the calyx and its substances have not been studied.

*Prunus dulcis* (Rosaceae), known as almond, has its centre of origin from Turkey [18,19]. This species reduced lettuce radicle and hypocotyl elongation to 5.7% and 6.7% of control, respectively, in this study. Amygdalin (D-mandelonitrile-beta-D-gentiobioside) is a cyanogenic glycoside present in kernels and seeds of *P*. *dulcis* [32]. Seeds from Rosaceae species contain relatively high amounts (0.1 –17.5 mg/g) of amygdalin compared to seeds from non-Rosaceae species (0.01–0.2 mg/g) [32]. *P. dulcis*, like other species belonging to Rosaceae, contains up to 3% amygdalin in flowers, leaves and barks, that produces glucose, benzaldehyde and hydrogen cyanide as toxins during hydrolysis. Only a few studies have been reported about the allelopathic activity of this plant [33,34]. Although it contains amygdalin, there is no report about the allelopathy of *P*. *dulcis* seed.

*Prunus mahaleb* L., known as mahaleb cherry, is a wild member of the Rosaceae family *P. mahaleb*, which reduced lettuce radicle and hypocotyl elongation to 7.8% and 11.6% of control, respectively in this research. Turkey is described as micro centres for *Prunus* spp. [18,19]. The seed kernels have a high protein content and fixed oil (27–40%) and contain coumarins, tannins, and traces of hydrocyanic acid [35]. Coumarin, dihydrocoumarin, herniarin have been primarily found in the seed kernels [36]. The high content of coumarin (0.87 mg/g) has been determined in the kernels as the main class of metabolites [37]. Only a few studies have been reported about the bioactivity of this plant [38]. There is, however, no report on the allelopathic activity of *P. mahaleb* seed kernel. On the other hand, coumarin itself was found to be highly phytotoxic [39,40].

*Peganum harmala* L., belonging to the family Nitrariaceae, is a multipurpose medicinal plant that reduced lettuce radicle and hypocotyl elongation to 8.6% and 16.4% of control respectively in this study. Harmaline, harmine, harmalol, harmol, and tetrahydroharmine were identified and quantified as the main beta-carboline alkaloids in *P. harmala* extracts. Harmine and harmaline accumulated in dry seeds at 4.3% and 5.6% (*w/w*) respectively, harmalol at 0.6%, and tetrahydroharmine at 0.1% (*w/w*). The roots contained harmine and harmol at 2.0% and 1.4% (*w/w*), respectively [41]. There are some studies on the allelopathic activity of this plant [42,43,44]. *P. harmala* is one of the most commonly used medicinal and aromatic plants in folk medicine in Turkey [45].

*Rhus coriaria* L. (Anacardiaceae), known as sumac, is a perennial edible plant, which grows wild in Aegean, Mediterranean, Southeast, Central and Northern regions of Turkey. *R. coriaria* reduced lettuce radicle and hypocotyl elongation to 7.4% and 33.1% of control, respectively, in this study. Either the whole fruit or only the pericarp is used as a condiment in Turkey [46]. Gallic acid was determined as the primary phenolic acid in the extracts of *R. coriaria*, followed by cyanidin, peonidin, pelargonidin, petunidin, delphinidin glucosides and coumarates. *R. coriaria* also contains some organic acids including malic acid, citric acid, tartaric acid and fumaric acid [47,48]. Only a few studies have been reported about the allelopathic activity of this plant—extract from sumac leaves, shoot or stem prevented seed germination, reduced seedling growth of some weed species and showed antifungal activity [49,50,51]. There is no report about the allelopathy of *R. coriaria* fruit.

*Taraxacum officinale* L. (Asteraceae), also known as dandelion, reduced lettuce radicle and hypocotyl elongation to 7.1% and 45.5% of control, respectively. It contains flavonoids, including luteolin, apigenin, isoquercitrin, caffeic acid and chlorogenic acid, and also terpenoids, triterpenes and sesquiterpenes [52]. Only a few studies have reported about the allelopathic activity of this plant. The water extracts from leaves and roots of *T. officinale* showed an inhibitory effect on germination and initial growth of some selected weed species [53,54]. There is, however, no report about the allelopathy of *T. officinale* seed.

*Terminalia chebula* Retz. is a flowering evergreen tree belonging to the Combretaceae family and this species respectively reduced lettuce radicle and hypocotyl elongation to 7.1% and 41.0% of the control. It grows wild in tropical and sub-tropical diverse climatic conditions. Different classes of bioactive natural products in *T. chebula* include tannins, flavonoids, flavins, terpenoids, steroids, various phenols, functionalized aliphatic molecules and their glycosides [55]. *T. chebula* fruit is rich in tannic acid. The major constituents of tannic acid are chebulic acid, chebulagic acid, corilagin and gallic acid [56]. Hydroquinone, gentisic acid, vanillic acid, syringic acid and trans-ferulic acid were determined in the bark and leaf of *T. chebula* [57]. Only a few studies have been reported about the allelopathic activity of this plant. Leaf litter samples of *T. chebula* showed a strong inhibitory effect on *Lactuca sativa* by using the sandwich method [58]. The aqueous extracts of dried leaves, fruit pulp, the leaf and bark extracts of *T. chebula* inhibited the germination and radicle elongation of test crops [57,59,60]. However, there is no report about the allelopathy of *T. chebula* seed.

The results presented now provide benchmark information for future research. The allelopathic effect of plants is of particular interest in sustainable weed management. The species with significant plant growth inhibitory potentials are candidate plants for subsequent studies, which might be about isolation and identification of potent allelochemicals to find new compounds for bioherbicides or utilization of candidate species as mulching materials or ground cover crops [61,62,63,64,65,66].

The species with significant plant growth inhibitory potentials in this study are medicinal plants. Medicinal plants are used widely in folk medicine and essential commercial products in Turkey and can also be used as resource material for research in allelopathy. Bioactive compounds with medicinal properties could act as allelochemicals, and medicinal plants showed relatively strong allelopathic activity [2,12,13,14,46,67,68]. Many potent allelochemicals have been isolated from medicinal plants in recent years [30,31,69,70,71]. *Peganum harmala*, *Lavandula stoechas*, *Silybum marianum*, and *Nigella sativa* are some of the most common medicinal plants used in traditional medicine in Turkey [45], which showed significant plant growth inhibitory effects on test plant in this screening. At the end of the first broad screening results indicated that some medicinal species showed strong plant growth inhibitory activity.

## 4. Materials and Methods 

### 4.1. Material (Plant Samples)

Dried samples of plant species (roots, leaves, stems, seeds, flowers, calyxes, fruits and barks) were supplied as material from herbalists and traditional medicinal drug shops located in different cities of Turkey. The plant material mostly consisted of native species such as herbs, spices, crops, weeds, medicinal and aromatic plants. It was targeted mainly to collect common plants as material from herbalists. The primary reason for collecting samples from these sources was to cover more samples, as much as possible, during this first broad screening. Herbalists offer opportunities to gather a large number of dry plant samples that grow wild or as broadly cultivated plants in the country. Therefore, samples were classified as cultivated or wild as mentioned in Table S1. Materials for this research are commonly sold and kept by herbalists according to related regulations. The samples were placed in separate air-tight bags and kept until further use in the laboratory.

### 4.2. Method (Sandwich Method)

In this research, the collected dry materials were assayed for their potential allelopathic activity by the sandwich method [2,72], developed to evaluate potential allelopathy through leachates [73]. The method has been adopted [2,12,15] widely to screen large numbers of plants and successfully determined the allelopathic activities of plant materials under laboratory conditions. The method is a reliable, sensitive and less time-consuming bioassay method [2,12,15,16,17,72,73]. It has been used primarily for the screening of medicinal plants [2,12,13,14,68]. A dry sample of 10 or 50 mg was placed into six-well multi-dish plastic plate (35 mm × 18 mm, Thermo Fisher Scientific Inc., Suzhou, China). Commercially available agar (Nacalai Tesque, Kyoto, Japan) was prepared as 0.75% (*w/v*) as the growth medium. By using a pipette, 5 mL agar was poured into the six-well plate in two successions. Plant samples were placed between the two agar layers like a sandwich. Five seeds of the test plant lettuce (*Lactuca sativa* variety Legacy, Takii Seed Co. Ltd, Kyoto Japan) were placed on solidified agar. Lettuce seeds were used for the bioassay experiment as a test plant due to its germination reliability, uniform and rapid growth and sensitivity towards allelochemicals [67,74,75]. Each plate was sealed with the plastic tape to prevent dehydration, wrapped by aluminium foil and incubated (Incubator, NTS Model MI-25S, Nihon Techno Service Co. Ltd., Ushiku, Japan) under dark conditions for 72 h at 22 °C. Controls were prepared as described above without the dry plant material. After incubation, the lengths of hypocotyls and radicles were measured by millimetre paper with 1 mm accuracy and expressed as a percentage of the control. Treatments of 10 and 50 mg were replicated three times for each sample, and the means were expressed as a percentage of the control.

The radicle and hypocotyl elongation percentages of lettuce seedling of 10 and 50 mg sample treatment were used as data for the statistical approach. The means (M), standard deviations (SD) and the standard deviations variances (SDV) using bell curve for Microsoft Excel by a special program (from Social Survey Research Information Co. Ltd) were calculated. The concept of the SDV, which has been used by previous studies [2,12,13,14,16,17,72], was applied for the evaluation of allelopathic activity among samples. The criterion of SDV was estimated for the range of significant effects of the species. Criteria indices were set as * = M − 0.5 (SD), ** = M − 1.0 (SD), *** = M − 1.5 (SD), **** = M − 2.0 (SD), ***** = M − 2.5 (SD), which indicate the radicle and hypocotyl elongation rate. The total number of criteria indices for a sample is defined as total score to provide a unique index for ranking all samples and shown in Table 1.

## 5. Conclusions

This study is the first comprehensive report on the screening of a large number of Turkish plant species by bioassay for potential allelopathic activity. The sandwich method results of 126 species from 55 families were presented in this report providing benchmark information reflecting the allelopathic potential of samples. Through this screening, the strong inhibitory activities of several species were determined and most of them are medicinal plants. The results of this study have shown that *H. sabdariffa*, *P. dulcis*, *R. coriaria* and *P. mahaleb* are new potential allelopathic candidates for further exploits in weed control. The results of the present study are new findings that draw attention to possible allelopathic potentials of Turkish plants, especially medicinal ones. 

## Figures and Tables

**Table 1:** The radicle and hypocotyl elongation percentages of lettuce seedlings grown on agar gel containing plant materials tested by sandwich method. Overview of top 30 most inhibitory plants.

Species [Used Part *]	Radicle Elongation (% of Control)				Hypocotyl Elongation (% of Control)				Total Score of *
	10 mg	Crit.	50 mg	Crit.	10 mg	Crit.	50 mg	Crit.	
*Hibiscus sabdariffa* L. [C]	3.2	*****	5.1	**	6.6	*****	3.3	***	15
*Prunus dulcis* (Mill.) D.A. Webb [S]	5.7	****	0.0	***	6.7	*****	0.0	***	15
*Taraxacum officinale* (L.) Weber [S]	7.1	****	4.0	**	45.5	***	11.9	***	12
*Terminalia chebula* Retz. [S]	7.1	****	5.7	**	41.0	***	13.0	***	12
*Rhus coriaria* L. [Fr]	7.4	****	3.2	**	33.1	****	10.6	***	13
*Prunus mahaleb* L. [S]	7.8	****	3.1	**	11.6	*****	0.0	***	14
*Peganum harmala* L. [S]	8.6	****	4.2	**	16.4	*****	4.9	***	14
*Punica granatum* L. [Fl)	24.5	***	8.3	**	56.4	**	45.3	*	8
*Alcea rosea* L. [L, Fl]	27.3	***	16.4	*	57.3	**	43.6	*	7
*Lavandula stoechas* L. [L, Fl]	27.4	***	1.9	**	81.5	*	0.5	***	9
*Silybum marianum* (L.) Gaertn. [S]	27.7	***	29.9		60.8	**	91.0		5
*Nigella sativa* L. [S]	27.8	***	14.0	**	15.1	*****	11.7	***	13
*Pinus brutia* Tenore [L]	31.4	**	9.0	**	92.7		50.0	*	5
*Vitis vinifera* L. [L]	32.9	**	11.7	**	66.6	**	44.5	*	7
*Liquidambar orientalis* L. [G]	36.0	**	3.8	**	40.7	***	1.2	***	10
*Camellia sinensis* (L.) Kuntze [L]	36.2	**	13.5	**	87.9		53.6	*	5
*Malva sylvestris* L. [L, Fl]	37.0	**	15.4	*	83.8		63.9		3
*Liquidambar orientalis* L [B]	37.5	**	6.1	**	46.8	***	16.8	***	10
*Citrus sinensis* (L.) Osbeck [P]	37.9	**	28.8		56.3	**	43.7	*	5
*Tribulus terrestis* L. [L]	39.5	**	18.6	*	104		91.9		3
*Capsella bursa-pastoris* (L.) Medik. [L]	40.7	**	26.5	*	82.3	*	74.9		4
*Papaver rhoeas* L. [L]	41.9	**	28.8		128		102.3		2
*Raphanus sativus* L. [S]	42.1	**	26.5	*	63.5	**	84.1		5
*Matricaria chamomilla* L. [L, Fl]	42.3	**	23.0	*	109		93.6		3
*Capsicum annum* L. [Fr]	44.5	*	18.9	*	85.9		70.5		2
*Corylus avellana* L. [L]	44.5	*	44.4		83.9		90.1		1
*Brassica napobrassica* (L.) Mill. [S]	49.8	*	24.7	*	65.2	**	41.5	*	5
*Ocimum basilicum* L. [L]	50.2	*	29.7		98.3		78.3		1
*Valeriana officinalis* L. [R]	50.3	*	6.5	**	75.0	*	24.7	**	6
*Artemisia absinthium* L. [L]	52.1	*	37.3		102		86.3		1
Mean, M	69.5		42.3		99.6				
Standard Deviation, SD	26.0		27.9		32.8				
M−0.5 × SD	56.5	*	28.4	*	83.2	*	57.8	*	
M−1.0 × SD	43.5	**	14.5	**	66.8	**	38.0	**	
M−1.5 × SD	30.5	***	0.6	***	50.4	***	18.2	***	
M−2.0 × SD	17.5	****			34.0	****			
M−2.5 × SD	4.5	*****			17.6	*****			

* Abbreviations: B = Bark, C = Calyx, Fl = Flower, Fr = Fruit, G = Gum, L = Leaf, P = Peel, R = Root, S = Seed.

**Table 2:** Taxonomic diversity of the evaluated species collected from Turkey.

Plant Family	Number of Species	Percent (%) of Species	Number of Samples	Percent (%) of Samples
Lamiaceae	15	11.9	15	10.4
Asteraceae	14	11.1	14	9.7
Rosaceae	11	8.7	13	9.0
Apiaceae	9	7.1	11	7.6
Brassicaceae	5	4.0	5	3.5
Fabaceae	5	4.0	6	4.2
Malvaceae	5	4.0	5	3.5
Cupressaceae	3	2.4	3	2.1
Papaveraceae	3	2.4	3	2.1
Poaceae	3	2.4	5	3.5
Other 45 *	53	29.4	64	44.4
Total	126	100	144	100

Other * means plant families with one or two species.

**Table 3:** Comparison of results of this study to previous reports.

References	Sample Number	M	SD	Country
[2]	239	68.3	25.7	Japan
[12]	170	60.4	21.3	Peru
[13]	251	71.5	21.0	China, Japan
[14]	145	65.7	21.9	Malaysia
Previous Studies	805	67.2	-	-
Current Study	144	69.5	26.0	Turkey
Summary	949	67.5	-	-

## Supplementary Materials

The following are available online at https://www.mdpi.com/2223-7747/8/12/532/s1, Table S1. The radicle and hypocotyl elongation percentages of lettuce seedlings grown on agar gel containing plant materials tested by Sandwich Method.
