# Supplementary material for: First Broad Screening of Allelopathic Potential of Wild and Cultivated Plants in Turkey
Source: Plants (Basel). 2019 Nov 21;8(12):532. doi: 10.3390/plants8120532 (PMC6963494; doi:10.3390/plants8120532)
Supplement: Supplementary file 1 [file plants-08-00532-s001.pdf]

plants MDPI

Table S1. Radicle and hypocotyl elongation percentages of lettuce seedlings grown on agar gel containing plant materials tested by Sandwich Method

**Family**	**Species**	**$R_{10\ mg}$ (%)**	**Criteria at $R_{10\ mg}$**	**$H_{10\ mg}$ (%)**	**Criteria at $H_{10\ mg}$**	**$R_{50\ mg}$ (%)**	**Criteria at $R_{50\ mg}$**	**$H_{50\ mg}$ (%)**	**Criteria at $H_{50\ mg}$**	**Used Part[*]**	**Total Score**	**Cult.[**] /Wild**
Malvaceae	*Hibiscus sabdariffa* L.	3.2	*****	6.6	*****	5.1	**	3.3	***	C	15*	Cult.
Rosaceae	*Prunus dulcis* (Mill.) D.A. Webb	5.7	****	6.7	*****	0.0	***	0.0	***	S	15*	Cult.
Asteraceae	*Taraxacum officinale* (L.) Weber	7.1	****	45.5	***	4.0	**	11.9	***	S	12*	Wild
Combretaceae	*Terminalia chebula* Retz.	7.1	****	41.0	***	5.7	**	13.0	***	S	12*	Cult.
Anacardiaceae	*Rhus coriaria* L.	7.4	****	33.1	****	3.2	**	10.6	***	Fr	13*	Wild
Rosaceae	*Prunus mahaleb* L.	7.8	****	11.6	*****	3.1	**	0.0	***	S	14*	Wild
Nitrariaceae	*Peganum harmala* L.	8.6	****	16.4	*****	4.2	**	4.9	***	S	14*	Wild
Lythraceae	*Punica granatum* L.	24.5	***	56.4	**	8.3	**	45.3	*	Fl	8*	Cult.
Malvaceae	*Alcea rosea* L.	27.3	***	57.3	**	16.4	*	43.6	*	L, Fl	7*	Cult.
Lamiaceae	*Lavandula stoechas* L.	27.4	***	81.5	*	1.9	**	0.5	***	L, Fl	9*	Wild
Asteraceae	*Silybum marianum* (L.) Gaertn.	27.7	***	60.8	**	29.9		91.0		S	5*	Wild
Ranunculaceae	*Nigella sativa* L.	27.8	***	15.1	*****	14.0	**	11.7	***	S	13*	Wild
Pinaceae	*Pinus brutia* Tenore	31.4	**	92.7		9.0	**	50.0	*	L	5*	Wild
Vitaceae	*Vitis vinifera* L.	32.9	**	66.6	**	11.7	**	44.5	*	L	7*	Cult.
Altingiaceae	*Liquidambar orientalis* L.	36.0	**	40.7	***	3.8	**	1.2	***	G	10*	Wild
Theaceae	*Camellia sinensis* (L.) Kuntze	36.2	**	87.9		13.5	**	53.6	*	L	5*	Cult.
Malvaceae	*Malva sylvestris* L.	37.0	**	83.8		15.4	*	63.9		L, Fl	3*	Wild
Altingiaceae	*Liquidambar orientalis* L.	37.5	**	46.8	***	6.1	**	16.8	***	B	10	Wild
Rutaceae	*Citrus sinensis* (L.) Osbeck	37.9	**	56.3	**	28.8		43.7	*	P	5*	Cult.
Zygophyllaceae	*Tribulus terrestis* L.	39.5	**	104		18.6	*	91.9		L	3*	Wild

Table S1. continued

**Family**	**Species**	**R$_{10\ mg}$ (%)**	**Criteria at R$_{10\ mg}$**	**H$_{10\ mg}$ (%)**	**Criteria at H$_{10\ mg}$**	**R$_{50\ mg}$ (%)**	**Criteria at R$_{50\ mg}$**	**H$_{50\ mg}$ (%)**	**Criteria at H$_{50\ mg}$**	**Used Part***	**Total Score**	**Cult. ** /Wild**
Brassicaceae	*Capsella bursa-pastoris* (L.) Medik.	40.7	**	82.3	*	26.5	*	74.9		L	4*	Wild
Papaveraceae	*Papaver rhoeas* L.	41.9	**	128		28.8		102.3		L	2*	Wild
Brassicaceae	*Raphanus sativus* L.	42.1	**	63.5	**	26.5	*	84.1		S	5*	Wild
Asteraceae	*Matricaria chamomilla* L.	42.3	**	109		23.0	*	93.6		L, Fl	3*	Wild
Solanaceae	*Capsicum annum* L.	44.5	*	85.9		18.9	*	70.5		Fr	2*	Cult.
Betulaceae	*Corylus avellana* L.	44.5	*	83.9		44.4		90.1		L	1*	Wild
Brassicaceae	*Brassica napobrassica* (L.) Mill.	49.8	*	65.2	**	24.7	*	41.5	*	S	5*	Cult.
Lamiaceae	*Ocimum basilicum* L.	50.2	*	98.3		29.7		78.3		L	1*	Cult.
Caprifoliaceae	*Valeriana officinalis* L.	50.3	*	75.0	*	6.5	**	24.7	**	R	6*	Wild
Asteraceae	*Artemisia absinthium* L.	52.1	*	102		37.3		86.3		L	1*	Wild
Asteraceae	*Artemisia dracunculus* Ledeb.	52.3	*	112		17.1	*	48.3	*	L	3*	Cult.
Apiaceae	*Foeniculum vulgare* (L.) Mill.	52.4	*	85.2		33.7		65.8		S	1*	Wild
Fabaceae	*Vicia ervilia* (L.) Wild.	53.0	*	83.2	*	27.3	*	25.8	**	S	5*	Wild
Asteraceae	*Calendula officinalis* L.	54.3	*	74.7	*	39.4		67.7		L, Fl	2*	Wild
Apiaceae	*Cuminum cyminum* L.	54.6	*	89.6		35.5		70.4		S	1*	Cult.
Linaceae	*Linum usitatissimum* L.	54.8	*	111		23.3	*	64.6		S	2*	Cult.
Fabaceae	*Trigonella foenum-graecum* L.	55.4	*	99.4		25.0	*	71.2		L	2*	Wild
Apiaceae	*Daucus carota* L.	55.5	*	83.5		38.4		78.2		S	1*	Cult.
Apiaceae	*Angelica sinensis* (Oliv.) Diels	56.4	*	92.2		33.6		75.2		L	1*	Cult.
Fabaceae	*Trigonella foenum-graecum* L.	56.4	*	89.0		28.8		62.1		S	1*	Wild

Table S1. continued

**Family**	**Species**	**$R_{10\,mg}$ (%)**	**Criteria at R_{10mg}**	**$H_{10\,mg}$ (%)**	**Criteria at $H_{10\,mg}$**	**$R_{50\,mg}$ (%)**	**Criteria at $R_{50\,mg}$**	**$H_{50\,mg}$ (%)**	**Criteria at $H_{50\,mg}$**	**Used Part[*]**	**Total Score**	**Cult.[**] /Wild**
Rosaceae	*Rosa canina* L.	57.0		120		26.9	*	94.7		Fr	1*	Wild
Poaceae	*Avena sativa* L.	57.4		77.3	*	44.5		77.8		H, St	1*	Wild
Betulaceae	*Corylus avellana* L.	57.9		100		31.1		90.0		S		Wild
Lamiaceae	*Mentha longifolia* L.	58.5		90.0		31.9		66.6		L		Wild
Asteraceae	*Solidago canadensis* L.	59.4		126		46.3		101		L, Fl		Wild
Vitaceae	*Vitis vinifera* L.	59.6		108		47.5		79.6		Fr		Cult.
Cupressaceae	*Juniperus drupacea* Labill.	59.6		60.2	**	65.8		52.8	*	R	3*	Wild
Apiaceae	*Anethum graveolens* L.	59.7		83.6		21.5	*	61.9		L	1*	Wild
Anacardiaceae	*Rhus coriaria* L.	60.8		89.1		83.4		108		S		Wild
Solanaceae	*Solanum lycopersicum* L.	61.5		119		53.7		87.7		Fr		Cult.
Apiaceae	*Pimpinella anisum* L.	61.6		63.0	**	16.0	*	8.3	***	S	6*	Wild
Lamiaceae	*Ocimum minimum* L.	62.1		134		29.9		77.8		L		Cult.
Brassicaceae	*Lepidium sativum* L.	62.3		94.8		22.2	*	44.1	*	S	2*	Wild
Adoxaceae	*Sambucus nigra* L.	62.6		101		37.3		75.8		L		Cult.
Cannabaceae	*Humulus lupulus* L.	63.3		96.8		13.0	**	9.8	***	L, Fl	5*	Wild
Papaveraceae	*Chelidonium majus* L.	63.5		112		23.2	*	59.3		L	1*	Wild
Cupressaceae	*Juniperus communis* L.	64.3		127		15.1	*	78.4		S	1*	Wild
Malvaceae	*Abelmoschus esculentus* (L.) Moench.	64.9		76.5	*	38.7		47.5	*	S	2*	Cult.
Lamiaceae	*Mentha pulegium* L.	65.6		98.8		1.4	**	0.7	***	L	5*	Wild
Urticaceae	*Urtica dioica* L.	65.6		142		36.6		121		L		Wild

Table S1. continued

Family	Species	$R_{10\ mg}$ (%)	Criteria at $R_{10\ mg}$	$H_{10\ mg}$ (%)	Criteria at $H_{10\ mg}$	$R_{50\ mg}$ (%)	Criteria at $R_{50\ mg}$	$H_{50\ mg}$ (%)	Criteria at $H_{50\ mg}$	Used Part*	Total Score	Cult.** /Wild
Asteraceae	*Arctium lappa* L.	65.8		96.1		31.2		80.6		L		Wild
Asteraceae	*Cota tinctoria* (L.) J.Gay	66.2		119		35.8		104		L, Fl		Cult.
Lamiaceae	*Perilla frutescens* (L.) Britton	66.6		115		30.9		98.8		L		Cult.
Plantaginaceae	*Plantago lanceolata* L.	66.7		128		31.7		105		L		Wild
Fagaceae	*Castanea sativa* Mill.	66.8		57.1	**	15.9	*	49.4	*	Fl	4*	Wild
Lamiaceae	*Satureja thymbra* L.	67.0		132		47.8		76.8		L		Wild
Poaceae	*Hordeum vulgare* L.	67.1		123		47.3		102		S		Wild
Myristicaceae	*Myristica fragrans* Houtt.	67.1		50.0	***	48.7		28.8	**	S	5**	Cult.
Primulaceae	*Primula vulgaris* Huds.	67.5		122		20.1	*	60.0		L, Fl	1*	Wild
Rosaceae	*Rubus sanctus* Schreb.	67.7		115		22.0	*	87.0		L	1*	Wild
Lamiaceae	*Origanum majorana* L.	67.9		101		29.6		65.5		L		Wild
Orchidaceae	*Orchis militaris* L.	69.4		115		51.8		124		R		Wild
Brassicaceae	*Sinapsis alba* L.	70.8		73.2	*	20.8	*	61.6		S	2*	Wild
Apiaceae	*Petroselinum crispum* (Mill.) Fuss	72.0		59.8	**	47.9		37.7	**	S	4*	Cult.
Rutaceae	*Citrus reticulata* Blanco	72.5		123		51.3		92.3		L		Wild
Asteraceae	*Carthamus tinctorius* L.	73.2		130		44.5		114		Fl		Wild
Apiaceae	*Petroselinum crispum* (Mill.) Fuss	73.4		133		32.0		86.5		L		Cult.
Pinaceae	*Pinus brutia* Tenore	75.0		79.7	*	20.0	*	22.8	**	G	4*	Wild
Urticaceae	*Urtica dioica* L.	75.1		117		32.4		87.2		S		Wild
Poaceae	*Zea mays* L.	75.3		86.6		45.5		72.4		S		Cult.

Table S1. continued

Family	Species	$R_{10\ mg}$ (%)	Criteria at $R_{10\ mg}$	$H_{10\ mg}$ (%)	Criteria at $H_{10\ mg}$	$R_{50\ mg}$ (%)	Criteria at $R_{50\ mg}$	$H_{50\ mg}$ (%)	Criteria at $H_{50\ mg}$	Used Part*	Total Score	Cult.** /Wild
Myrtaceae	*Myrtus communis* L.	76.6		107		22.2	*	81.3		L	1*	Wild
Asteraceae	*Helichrysum arenarium* (L.) Moench	77.7		115		57.5		107		L, Fl		Wild
Juglandaceae	*Juglans regia* L.	78.0		112		52.2		70.3		L		Cult.
Pinaceae	*Pinus pinea* L.	79.4		126		58.8		122		S		Wild
Rhamnaceae	*Paliurus spina-christi* Mill	79.7		100		53.2		87.4		L		Wild
Moraceae	*Ficus carica* L.	80.1		127		62.5		133		L		Cult.
Lythraceae	*Punica granatum* L.	80.3		98.7		23.7	*	70.1		S	1*	Cult.
Gentianaceae	*Gentiana lutea* L.	80.4		68.9	*	22.0	*	48.6	*	L	3*	Wild
Apiaceae	*Apium graveolens* L.	80.8		82.3	*	37.8		31.4	**	S	3*	Wild
Onagraceae	*Epilobium angustifolium* L.	81.0		115		20.3	*	69.6		L	1*	Wild
Poaceae	*Zea mays* L.	81.2		113		47.4		108		Stg		Cult.
Betulaceae	*Corylus avellana* L.	81.3		128		55.0		138		Cp		Wild
Malvaceae	*Tilia tomentosa* Moench	81.5		103		41.9		95.5		L, Fl		Wild
Piperaceae	*Piper nigrum* L.	81.8		81.8	*	27.4	*	41.5	*	Fr	3*	Cult.
Asteraceae	*Cynara cardunculus* L.	81.9		159		44.4		125		L		Wild
Gentianaceae	*Centaurium erythraea* Rafn	83.0		99.3		16.7	*	53.1	*	L, Fl	2*	Wild
Fabaceae	*Glycyrrhiza glabra* L.	83.6		109		55.1		70.3		R		Wild
Apiaceae	*Anethum graveolens* L.	84.0		104		34.8		59.7		S		Wild
Lamiaceae	*Lavandula angustifolia* Mill.	84.1		72.7	*	0	***	0	***	Fl	7*	Wild
Rosaceae	*Crataegus monogyna* Jacq.	84.5		123		86.2		123		S		Wild

Table S1. continued

Family	Species	$R_{10\,mg}$ (%)	Criteria at $R_{10\,mg}$	$H_{10\,mg}$ (%)	Criteria at $H_{10\,mg}$	$R_{50\,mg}$ (%)	Criteria at $R_{50\,mg}$	$H_{50\,mg}$ (%)	Criteria at $H_{50\,mg}$	Used Part*	Total Score	Cult.** /Wild
Lamiaceae	*Melissa officinalis* L.	84.6		110.3		39.7		59.2		L		Wild
Cupressaceae	*Tetraclinis articulata* (Vahl) Mast.	84.7		85.6		98.5		79.8		G		Cult.
Rosaceae	*Malus domestica* Borkh.	84.7		83.1	*	72.2		93.1		Fr	1*	Cult.
Lamiaceae	*Thymus serpyllum* L.	85.2		63.6	**	43.3		26.0	**	L	4*	Cult.
Apocynaceae	*Nerium oleander* L.	85.3		125		81.7		129		L		Wild
Papaveraceae	*Papaver somniferum* L.	85.3		72.8	*	67.9		84.6		S	1*	Cult.
Platanaceae	*Platanus orientalis* L.	85.5		147		44.4		123		L		Wild
Verbenaceae	*Vitex agnus-castus* L.	86.7		118		65.6		115		S		Wild
Hypericaceae	*Hypericum perforatum* L.	87.5		178		33.7		98.5		Fl		Wild
Vitaceae	*Vitis vinifera* L.	87.7		96.0		65.1		91.4		S		Cult.
Fabaceae	*Phaseolus vulgaris* L.	88.6		110		32.6		72.6		S		Cult.
Elaeagnaceae	*Elaeagnus angustifolia* L.	89.5		113		31.1		85.2		L		Cult.
Rosaceae	*Crataegus monogyna* Jacq.	92.1		111		73.5		88.3		L		Wild
Asteraceae	*Achillea millefolium* L.	92.4		98.0		70.8		89.1		L		Wild
Lauraceae	*Persea americana* Mill.	92.4		141		48.0		112		L		Cult.
Betulaceae	*Corylus avellana* L.	93.9		101		101		157		Sh		Wild
Rosaceae	*Alchemilla vulgaris* L.	94.4		92.5		36.9		67.6		L		Wild
Santalaceae	*Viscum album* L.	95.1		138		17.2	*	32.9	**	L	3*	Wild
Asteraceae	*Echinacea purpurea* (L.) Moench	95.7		116		81.4		100		L, Fl		Cult.
Moraceae	*Morus alba* L.	95.7		132		68.6		130		L		Cult.
Apiaceae	*Coriandrum sativum* L.	96.9		132		87.6		131		S		Wild

Table S1. continued

Family	Species	$R_{10\ mg}$ (%)	Criteria at $R_{10\ mg}$	$H_{10\ mg}$ (%)	Criteria at $H_{10\ mg}$	$R_{50\ mg}$ (%)	Criteria at $R_{50\ mg}$	$H_{50\ mg}$ (%)	Criteria at $H_{50\ mg}$	Used Part*	Total Score	Cult.** /Wild
Lamiaceae	*Salvia fruticosa* Mill.	97.5		125.6		70.7		98.0		L		Wild
Ericaceae	*Calluna vulgaris* (L.) Hull	97.9		118		83.5		120		L		Wild
Lamiaceae	*Satureja hortensis* L.	100		97.5		44.5		54.3	*	L	1*	Wild
Lamiaceae	*Rosmarinus officinalis* L.	100		153		80.1		152		L		Wild
Myrtaceae	*Eucalyptus globulus* Labill.	100		80.5	*	40.4		40.6	*	L	2*	Cult.
Equisetaceae	*Equisetum telmateia* Ehrh.	101		146		91.4		153		L		Cult.
Rosaceae	*Rosa canina* L.	101		124		87.8		128		S		Wild
Rosaceae	*Prunus armeniaca* L.	101		175		88.8		161		S		Cult.
Boraginaceae	*Alkanna tinctoria* (L.) Tausch	101		109		102		111		L		Wild
Lauraceae	*Laurus nobilis* L.	102		156		44.7		97.8		L		Wild
Rosaceae	*Prunus avium* L.	102		94.5		51.5		76.1		St		Cult.
Lamiaceae	*Thymus vulgaris* L.	102		106		63.8		61.4		L		Cult.
Juglandaceae	*Juglans regia* L.	102		137		79.9		131		Fr		Cult.
Poaceae	*Avena sativa* L.	104		86.6		55.1		125		S		Wild
Rosaceae	*Sorbus aucuparia* L.	104		135		95.9		155		L		Wild
Anacardiaceae	*Pistacia lentiscus* L.	104		98.8		121		130		G		Wild
Fabaceae	*Ceratonia siliqua* L.	109		121		71.9		90.1		Fr		Wild
Oleaceae	*Olea europaea* L.	109		108		118		127		L		Wild
Alliaceae	*Allium cepa* L.	111		121		95.6		129		S		Cult.
Pteridaceae	*Adiantum capillus-veneris* L.	112		128		98.9		134		L		Wild

Table S1. Continued

Family	Species	$R_{10\ mg}$ (%)	Criteria at $R_{10\ mg}$	$H_{10\ mg}$ (%)	Criteria at $H_{10\ mg}$	R_{50mg} (%)	Criteria at $R_{50\ mg}$	$H_{50\ mg}$ (%)	Criteria at $H_{50\ mg}$	Used Part*	Total Score	Cult. **/Wild
Liliaceae	*Allium porrum* L.	112		152		117		156		S		Cult.
Rosaceae	*Cydonia oblonga* Mill.	114		137		83.5		145		L		Cult.
Cucurbitaceae	*Cucumis sativus* L.	118		162		65.6		105		S		Cult.
Mean, M		69.5		99.6		42.3		77.6				
Standard Deviation, SD		26.0		32.8		27.9		39.6				
M − 0.5 × SD		56.5	*	83.2	*	28.4	*	57.8	*			
M − 1.0 × SD		43.5	**	66.8	**	14.5	**	38.0	**			
M − 1.5 × SD		30.5	***	50.4	***	0.6	***	18.2	***			
M − 2.0 × SD		17.5	****	34.0	****							
M − 2.5 × SD		4.5	*****	17.6	*****							

The criteria *sign indicates the strength of inhibitory activity of 10 mg and 50 mg samples on the radicle elongation of lettuce as evaluated by using standard deviation. Increasing number of the criteria *sign mentions increasing inhibitory activity of samples. The values which are close to 0 means strong inhibitory activity of samples on the growth of lettuce seed, test plant.

$R_{10\ mg}$%: Radicle elongation percentage of lettuce seed with 10 mg sample treatment, $Radicle_{10\ mg}$%

$R_{50\ mg}$%: Radicle elongation percentage of lettuce seed with 50 mg sample treatment, $Radicle_{50\ mg}$%:

$H_{10\ mg}$%: Hypocotyl elongation percentage of lettuce seed with 10 mg sample treatment, $Hypocotyl_{10\ mg}$%:

$H_{50\ mg}$%: Hypocotyl elongation percentage of lettuce seed with 50 mg sample treatment, $Hypocotyl_{50\ mg}$%:

* Abbreviations: B = Bark, C = Calyx, Cp = Capsule, Fl =Flower, Fr = Fruit, G = Gum, H = Husk, L = Leaf, P = Peel, R = Root, S = Seed, Sh = Shell, St = Stem, Stg = Stigma.

**Cult. = Cultivated.
